# Biomaterials, Current Strategies, and Novel Nano-Technological Approaches for Periodontal Regeneration

**DOI:** 10.3390/jfb10010003

**Published:** 2019-01-02

**Authors:** Giorgio Iviglia, Saeid Kargozar, Francesco Baino

**Affiliations:** 1 Nobil Bio Ricerche Srl, 14037 Portacomaro d’Asti, Italy; giviglia@nobilbio.it; 2 Department of Modern Sciences and Technologies, School of Medicine, Mashhad University of Medical Sciences, Mashhad 917794-8564, Iran; kargozarsaeid@gmail.com; 3 Institute of Materials Physics and Engineering, Department of Applied Science and Technology, Politecnico di Torino, 10129 Torino, Italy

**Keywords:** dental materials, periodontal tissue engineering, dental implants, bioceramics, polymers, composites, bioactivity, nanotechnology, nanomaterials

## Abstract

Periodontal diseases involve injuries to the supporting structures of the tooth and, if left untreated, can lead to the loss of the tooth. Regenerative periodontal therapies aim, ideally, at healing all the damaged periodontal tissues and represent a significant clinical and societal challenge for the current ageing population. This review provides a picture of the currently-used biomaterials for periodontal regeneration, including natural and synthetic polymers, bioceramics (e.g., calcium phosphates and bioactive glasses), and composites. Bioactive materials aim at promoting the regeneration of new healthy tissue. Polymers are often used as barrier materials in guided tissue regeneration strategies and are suitable both to exclude epithelial down-growth and to allow periodontal ligament and alveolar bone cells to repopulate the defect. The problems related to the barrier postoperative collapse can be solved by using a combination of polymeric membranes and grafting materials. Advantages and drawbacks associated with the incorporation of growth factors and nanomaterials in periodontal scaffolds are also discussed, along with the development of multifunctional and multilayer implants. Tissue-engineering strategies based on functionally-graded scaffolds are expected to play an ever-increasing role in the management of periodontal defects.

## 1. Current Strategies in Periodontal Tissue Engineering 

The primary goal of periodontal tissue engineering is to regenerate the supporting tissues of the tooth. Tooth loss can be the result of trauma or periodontal diseases, including gingivitis, periodontitis, or age-related tissue decay. There are two distinct aims in periodontal tissue engineering strategies, which include the formation of new alveolar bone and connective tissue (cementum and periodontal ligament). If left untreated, periodontal disease invariably leads to the loss of the tooth, and it has been estimated that about 10% of the global population is affected by severe periodontitis [1]. Statistics have shown that above 300,000 dental implants are placed annually only in the USA, and this number is expected to further increase in the next decade [2,3,4]. Tooth replacement by using titanium dental implants is currently a routine procedure with a success rate of around 98%; however, it still suffers from some limitations. In this regard, it cannot be ignored that the positive outcome of a titanium screw insertion mainly depends on the quantity and quality of the patient’s alveolar bone which can support the implant in the extraction site [5]; hence, the development of suitable strategies for the regeneration of hard tissue is of paramount importance in dental surgery. In the last decades, the treatment of soft tissue defects has also gained increasing attention in order to prevent advanced forms of periodontal injuries related to mucogingival anomalies and better satisfy the aesthetic demands of patients [6]. 

**Figure 1:**
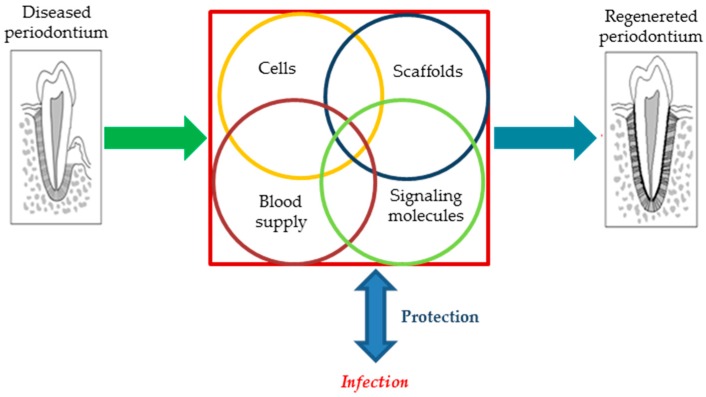
Schematic representation of the key parameters involved in the periodontal regeneration.

Periodontal regeneration is recognized as one of the primary clinical disciplines which has benefitted from the novel technologies based on tissue engineering [7]. Biomaterials, bioactive molecules (e.g., growth factors), and (stem) cells are the three critical factors behind the management of periodontal disease (see Figure 1). Current therapies for periodontal tissue repair and regeneration include conservative approach, radicular conditioning, bone grafting/substitution, and guided tissue regeneration (GTR), as well as a combination of the two last strategies. 

Conservative therapy includes the surgical debridement of the periodontal pathogenic microorganisms, mineralized deposits on the root surface, infected cementum as well as all the injured/necrotic tissue parts [8,9]. This procedure is fundamental to allow periodontal healing and commonly precedes any type of reconstructive strategy. This approach relies on the concept that patients’ fibroblasts from the epithelium are the cells possessing the fastest growth ability; thus, increasing the distance between the epithelial layer and the wound by surgery allows slower connective tissue formation and prevents fibroblasts from achieving the radicular surface before the osteogenic cells. The importance of debridement was first reported by Prichard [10] in the late 1950s and, since then, the results have always confirmed the need for reaching specific clinical conditions that include lack of inflammation as well as a minimized and under-controlled bacterial plaque. These situations are ideal for allowing the regeneration of the periodontal tissue [11,12,13]. 

As a direct result of periodontal disease or after a surgical procedure, the root surface could be directly exposed to the oral environment that is invariably full of various types of bacteria. The radicular surface is a suitable substrate for bacterial adhesion and biofilm formation, with the associated risk of the increased inflammatory response [14]. In these cases, chemical conditioners are often used to obtain a more biocompatible surface for healthy cells [8,15]. Citric acid, ethylenediaminetetraacetic acid (EDTA) and tetracycline are the most commonly-used acids, the role of which is to decontaminate the surface from bacteria and endotoxins. Furthermore, the surface of collagen fibers is exposed to the etching of detergents, thereby potentially providing a good condition for adhesion of cells that are responsible for tissue regeneration [8]. Despite the minor invasiveness, the actual superiority and clinical benefits of using non-surgical disinfection compared to surgical debridement still remains controversial [16]. 

The above-mentioned techniques alone promote periodontal repair rather than periodontal regeneration; a good repopulation of the defect site can be achieved by implanting bone grafting materials (after debridement) that stimulate new bone formation [17]. Tissue regeneration is often achieved by combining bone grafting materials and guided tissue membranes, with or without the use of specific growth factors. The use of stem cells is now under development and is considered as a more recent tactic [18,19], the potential of which could allow overcoming the well-known major drawbacks of most synthetic biomaterials, i.e., the lack in the induction of tissue formation. Despite the great promise of stem cells, there are still many safety, regulatory, and ethical issues concerning their clinical use. 

## 2. Inside Periodontal Regeneration—A More In-Depth Overview

At present, the majority of tissue-engineering strategies applied to treat periodontal diseases aim at regenerating the alveolar bone through the implantation of suitable scaffold-based constructs. Specifically, periodontal regeneration relies on four basic paradigms [20]:

(i) Implanted material (scaffold) acts as a three-dimensional (3D) template supporting new tissue growth; 

(ii) Cells are the primary building blocks of the reconstructive strategies of periodontal tissue thanks to their proliferation and differentiation;

(iii) Bioactive molecules (i.e., growth factors) promote cell activity that results in improved cell proliferation and differentiation;

(iv) The blood supply delivers oxygen, nutrients, and essential factors to tissue, and thus promotes the growth of newly formed tissue and helps to maintain homeostasis inside the 3D scaffold. 

The healing process of the periodontal tissue is traditionally achieved by tissue repair; however, the advent of tissue engineering and regenerative medicine provides new possibilities in this regard [7]. Based on the definition, repair process includes the healing of a wound by the growth of tissue that, however, may not fully restore the fine microstructure and, consequently, the function of the lost tissue [21,22]. Moving a step forward, regenerative medicine uses tissue-engineered constructs to restore the original architecture and function of the injured tissue [23]. Two main objectives are simultaneously pursued in the current periodontal tissue engineering approaches, including (i) the stimulation of the growth of the key surrounding tissues by applying barrier membranes and bone grafting materials and (ii) the prevention of the growth and proliferation of undesired cell types like epithelial cells [24]. 

The periodontium in its native form is comprised of various parts including alveolar bone, cementum, junctional epithelium, and a gingival connective attachment. This tissue is a result of the interaction between mesenchymal and epithelial cells in the embryo [25,26]. A series of independent and subsequent events need to complete the periodontal regeneration, which include osteogenesis, cementogenesis, and connective tissue formation [27]. It has been well documented that various physico-chemical and mechanical stimuli can cause appropriate responses by the cells during the healing process. The type of cells used in regeneration controls the quality of the healing process since they are responsible for repopulation of the wound first [24]. In the case of the periodontal regeneration, four distinct cell types compete—namely, periodontal ligament cells, alveolar bone cells, cementoblasts, and epithelial cells. Among these cells, the first three types are responsible for regenerating the periodontal tissue, while the last type—i.e., epithelial cells—are responsible for soft tissue regeneration. It is worth mentioning that the higher migration rate of epithelial cells (10 times faster) in comparison to the other periodontal cell types is the reason of observing the formation of the long junctional epithelium in the periodontal therapy [28]. Infiltration of epithelial cells inside the defect can promote repair by the formation of unusual architecture with a loss of function [29]. Therefore, guided tissue membranes are implanted to limit the infiltration of the epithelial cells [30]. If epithelial cells are ruled out from the wound, other cell types with regenerative potential are thus allowed to become established, and epithelial down-growth can be successfully prevented [24]. A combination of bone graft materials, promoting the migration and differentiation of osteoblast cells, and GTR is the most commonly-used approach for achieving an optimal periodontal regeneration [31,32]. Two reasons have been identified behind this synergic activity, i.e., the biological effects of bone grafts and the “Melcher hypothesis”, which explains the importance of cells used for the periodontal regeneration. According to this hypothesis, the origin of cells dictates the nature of the attachment in periodontal healing and the complete periodontal regeneration may be achieved when we apply cells with an origin from the periodontal ligament and the perivascular bone cells [8,33,34]. 

The biological actions induced by bone grafting materials can be divided into three interrelated healing processes: osteogenesis, osteoconduction, and osteoinduction [35]. The autologous bone graft is the ‘gold standard’ option to achieve osteogenesis [36]. In this case, the undifferentiated and pluripotent cells derived from the transplanted material differentiate into the bone-forming cell lineage, i.e., osteoblasts, which will form new bone [37]. Osteoconduction refers to the ability of a biomaterial to act a suitable surface for guiding the bone growth by pre-existing preosteoblasts/osteoblasts [35,38]. Osteoinduction is usually identified as a specific property of materials regarding the induction of the differentiation of the host osteoprogenitor cells into osteoblasts [39]. In this case, the graft material excludes the undifferentiated connective cells and just induces the differentiation and proliferation of osteoprogenitor ones, thus leading to new bone formation [35,40,41]. The Melcher hypothesis also points out the importance of barrier materials for enabling cell migration from the connective tissue to avoid the repair process [24,32,42]. Numerous previously performed animal studies clarified the efficacy of non-resorbable and resorbable membranes for GTR [32,42].

Three correlated steps are involved in the healing process of periodontal tissue injuries. The first step is the epithelization of the internal face of the flap that forms the so-called long epithelium attachment. The maturation of the connective tissue is the second step in which we observe the formation of the so-called connective attachment. The final step is related to the recovery of bone architecture and the periodontal ligament at the level of alveolar bone and in the deepest point of injury. The morphology of the structure of newly formed tissue indicates whether repair or regeneration took place [7]. In the case of repair, fibroblasts mostly are involved in the healing process instead of osteoblasts, thus the deposition of the osteoid matrix and new bone formation are inhibited [21,22]. On the other hand, regeneration involves the complete recovery of both the structure and the function of the periodontal tissue [7,43]. 

**Figure 2:**
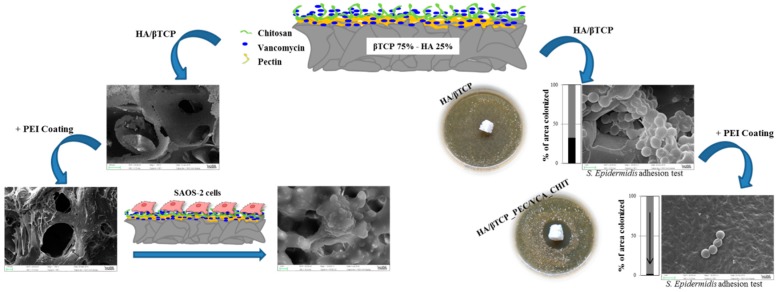
Schematic representation of an engineered ceramic scaffold developed for peri-prosthetic infection prevention. The functionalization of the ceramic scaffold with a pectin-chitosan hydrogel allows the control of antibiotic release, inhibits bacterial proliferation and biofilm formation, and promotes osteoblast proliferation. Optical and scanning electron microscopy pictures courtesy of Giorgio Iviglia.

Despite the implementation of this approach, the success of periodontal regeneration depends on the capacity to control local infections, that are caused by microbial pathogens contaminating periodontal wounds [44,45,46]. The removal of necrotic tissue or a zone with acute infection are often identified as the major complications of periodontal defects and are high-risk places for bacterial growth and re-infection of new tissue [47,48]. Hence, there is a need for implementing appropriate strategies to regenerate the periodontal tissue and restrain bacterial growth [49]. There are critical limitations in front of the currently-available strategies (e.g., conventional systemic antibiotic therapy) applied for reducing the risk of wound infection, including the systemic toxicity in the body which can lead to the need of hospitalization for monitoring [50,51,52,53]. In order to overcome these problems, several research groups are working on advanced systems able to release low dosage of antibiotics directly in situ (see Figure 2) [54,55,56,57,58,59,60]. Promising approaches for avoiding bacterial adhesion and minimizing the side effects of systemic antibiotics include the coating of titanium implants with antiseptic molecules, the implant modification with functional groups exerting a bactericidal action, and the use of specific signaling molecules which selectively act as bactericidal substances [55,61,62,63].

## 3. Biomaterials for Periodontal Tissue Engineering 

Bone grafting materials are widely used in periodontal tissue engineering in order to augment the alveolar bone and provide adequate mechanical support for a future, permanent implant. They include materials of biological (e.g., osseous transplants) or synthetic origin and are made available to dental surgeons in different forms and shapes, such as particles, 3D scaffolds, injectable pastes or composites in combination with polymeric materials. Given that microbial infections play a crucial role in the evolution of periodontal diseases and can have a devastating effect, in the last years some attempts to develop new dental materials with antibacterial properties have been carried out in order to control the infection and increase the implant success rate.

### 3.1. Bone Transplant Materials

#### 3.1.1. Autografts 

Autologous grafts are currently considered the ‘gold standard’ solution for bone replacement [64]. Autografts are tissue portions transplanted from one part of the body to another in the same patient. If the alveolar bone defect is small, the autologous material is usually harvested intraorally from the extraction sockets, edentulous ridges, ramus, symphysis, tuberosity, or from the surrounding buccal plate. In the presence of large bone defects, autografts are typically obtained from extra-oral areas, such as the iliac crest, tibia, or skull; invariably, this latter approach is more stressful to the patient due to the need for a second-site surgery. The implant survival rate for autologous bone grafts was reported to vary in the range of 76–100%, with the worse result for iliac crest bone compared to calvarial bone [65,66,67,68]. Other studies, however, reported that the results obtained with intraoral grafts were similar to those observed with extra-oral sources [67,69]. A robust comparison among all these data is not possible due to donor site variability and often unpredictable complications; hence, the debate about the ‘optimal’ autologous material still lingers on and, at present one cannot claim that a particular technique is clearly superior to another [70]. 

The significant advantages of using autografts are that they are osteogenic, prevent disease transmission and are relatively inexpensive. Clinical tests generally showed excellent periodontal regeneration with the formation of new cementum; for example, Schallhorn and colleagues used iliac crest grafts for the management and treatment of infrabony defects with good results (up to 4-mm bone healing) [71]. Major drawbacks include the need for a second surgery and the risk of possible complications at the harvesting site, such as infection and postoperative pain [72]. Furthermore, the limited availability of autologous material is an additional issue that makes this approach less attractive to treat large injuries.

#### 3.1.2. Allografts 

An allogenic graft is a biologic material harvested from a donor of the same species with a different genotype. The key advantage of allografts is the elimination of a second surgical procedure and tissue resource constraints [6]. This type of graft is usually available in tissue banks that process the donor (cadaver) tissues; depending on how the allografts are treated, the tissues can be divided into freeze-dried bone allografts (FDBAs) and decalcified freeze-dried bone allografts (DFDBAs). Allografts have the significant disadvantages to potentially elicit a foreign body immune response and carry the risk of disease transmission, albeit a robust safety and regulatory system has been developed in the last years at the international level [73,74,75]. Despite the mentioned limitations, the researchers and clinicians have recognized allografts as relatively high reliable sources for reconstructive applications since they can serve as osteoconductive or even osteoinductive materials based on the remaining proteins into their matrix [76]. While decalcification process of allografts can result in exposing bone morphogenetic proteins (BMPs) that are osteoinductive molecules for bone regeneration, this procedure is the main reason for the high resorption rate of allografts as compared to autografts, which can lead to less effective scaffolding properties. 

Several studies compared the clinical outcomes associated with FDBAs and DFDBAs. Yukna et al. performed animal studies in which both FDBAs and DFDBAs were placed into surgically-created infrabony defects and evaluated by histological analysis [77]. After three months, FDBAs showed higher new bone formation than DFDBAs. Altiere et al. [78] and Blumenthal et al. [79] also reported excellent results as regards new bone formation after using FDBAs. The high variability of these types of grafts was also pointed out: Mellonig showed that DFDBA exhibited similar bone-filling percentage compared to FDBA in one study [80] while observing higher bone-filling percentage in a previous study where FDBA was mixed with autologous material [81]. Another disadvantage of such an approach is the high material cost compared to other options like xenografts [82]. 

#### 3.1.3. Xenografts

Xenogenic materials are bone substitutes obtained from other species (typically bovine and porcine grafts) and transplanted into humans. In the past, storage of these grafts in tissue banks was often preferred as it is possible to extract larger amounts of bone from animals with a specific microstructure as compared to auto- and allografts. Since the main disadvantage of xenografts is their antigenicity, animal tissues should be carefully processed to remove all organic components. There are many commercial products based on this process, such as Geistlich BioOss^®^ particles (Geistlich Pharma, Wolhusen, Switzerland) from the bovine source, which is considered one of the reference products in dental applications worldwide.

However, in spite of the positive results obtained from studies conducted on xenografts, it cannot be ignored that the rate of graft resorption and bone regeneration with these materials might be unpredictable [83]. Taschieri et al. observed a periodontal healing rate of 78% in patients implanted with bovine-derived bone grafts after a one-year follow-up but did not detect any difference as compared to non-treated lesions where the only debridement was carried out [84]. In another study, eight infrabony defects were treated with xenografts, and the results showed that seven defects successfully healed with actual bone regeneration, while one defect healed by the growth of non-osseous tissue [85]. 

Being stored in tissue banks and made promptly available, xenografts are advantageous over autografts as they require a single surgical procedure; on the other hand, some people might refuse to be implanted with these animal-derived materials due to ethical and religious concerns.

### 3.2. Need for Alternatives to Bone Transplantation 

The osseous grafts described above suffer from different disadvantages such as the need for second-site additional surgery (autografts), the risk of disease transmission (allograft and xenografts), limited availability (autografts), and unpredictable results (xenografts). In recent decades, man-made biocompatible materials have gained increasing interest thanks to their attractive characteristics, including controlled degradation, osteoconductivity, and/or osteoinductivity, customized mechanical properties, and relatively easy shapability to match the defect dimensions [86]. Non-transplant materials used for periodontal tissue regeneration typically belong to the classes of ceramics and polymers or can be the result of their mixture [64,87]. Bioceramics include calcium phosphates, calcium sulfate, and bioactive glasses [88,89]. Polymers can be of natural (e.g., modified polysaccharides, polypeptides) or synthetic origin (e.g., poly(glycolic acid), poly(L-lactic acid)). Natural and synthetic polymers are often combined with ceramics for obtaining composite biomaterials with superior osteoinductive properties, enhanced mechanical performance, tunable degradation rate, and better cell adhesion [64,86,90]. 

### 3.3. Bioceramics 

#### 3.3.1. Calcium Phosphates

Calcium phosphate materials have a long history and the early studies about their biological significance date back to 1920 [91]. Since then, a huge amount of knowledge and data related to their chemistry, formulations, and properties has been produced and consolidated [92,93,94]. Calcium phosphates are very popular in bone and dental tissue engineering applications due to their compositional affinity with the mineral phase of natural bone. In general, these materials tend to induce a biological response which is similar to that generated during bone remodeling, i.e., resorption of the old bone and, in parallel, the formation of new bone. Since calcium phosphate grafts are chemically similar to the natural bone, their degradation products are non-toxic and can be naturally metabolized by mammals [95]. Implantable calcium phosphates are available as solid pieces, 3D porous scaffolds, granules, and pastes or cements. Hydroxyapatite (HA), tricalcium phosphates, and their different combinations are recognized as the most commonly-used calcium phosphates in periodontal reconstructive strategies. 

HA (Ca_10_(PO_4_)_6_(OH)_2_)) is the most abundant inorganic component of natural bone (around 65% of its inorganic phase) and has found a lot of applications as bone filler in the clinical practice. Many studies have been focused on the interaction between HA implants and bone [96,97,98,99,100]. A direct chemical bond was reported to form between osseous tissue and HA graft, which gives rise to a kind of bone matrix on the implant surface. This newly-formed matrix is composed of both globular mineral deposits and an organized network of collagen fibers, which may enhance the interfacial bonding [101]. Osteoblasts can attach on the HA surface and start forming mineralized osteoid, which then matures into fully mineralized bone [102]. Apatite crystals—which may also incorporate other ions like carbonate groups—appear on the surface of implanted HA grafts and exhibit a nano-crystalline morphology mimicking the biological apatite of the alveolar bone [103]. HA is osteoconductive but has a very slow degradation rate which limits its use alone [104]. 

Due to this reason, the interest in tricalcium phosphate (TCP) materials has rapidly increased in recent years. The degree of solubility mainly depends on the Ca-to-P ratio (the rate of dissolution increases with decreasing Ca/P ratio) as well as on the crystallographic structure, thus producing the following progression: HA < β-TCP ≪ α-TCP). 

The most attractive TCP phase in biomedicine is the β one, which has clearly shown good biocompatibility and osteoconductive properties [90]. Interestingly, β-TCP exhibits degradation kinetics comparable to the rate of new bone growth and regenerative properties similar to those of autologous bone grafts [105]. However, β-TCP has poor mechanical properties and, therefore, its use as implant material is typically recommended only in low-bearing applications and in particle form. Furthermore, the resorption rate could be too fast in very large bone defects. β-TCP resorption in vivo is based on two main mechanisms, i.e., the dissolution occurs due to the lixiviation by biological fluids or the action of osteoclast cells; a coexistence of the two was also hypothesized [106]. It was also shown that, as dissolution proceeds, the osteoclast activity decreases over time due to the high amount of calcium ions released into the implant surroundings (modulatory effect on cell behavior) [107,108]. The dissolution rate of the material depends on many parameters such as the sintering process, micro- and macro-porosity, and purity of the raw material [109]. A valuable strategy to control the degradation rate is to produce multiphasic ceramics by mixing different calcium phosphates according to a ratio which fits the needed characteristics [110,111]. 

A suitable grafting material for periodontal tissue engineering should be osteoconductive, be able to sustain the load applied on the defect site as new bone grows, and safely dissolve without producing any toxic ionic species for the surrounding tissue [64,112,113]. The balance between HA and β-TCP is a crucial point to obtain adequate mechanical strength, suitable degradation kinetics, and osteointegration in biphasic calcium-phosphate ceramics. Several studies were performed in order to assess the best HA-to-β-TCP ratio, but the results are difficult to robustly compare since many variables affect the conclusions from one to another group of researchers [110,114,115]. For example, it has been clarified that the degradation rate, as well as the mechanical and the biological properties, are extensively controlled by the sintering parameters, the mixing method, the raw material source as well as the final shape of the product (e.g., powders, granules, paste, 3D scaffold, and so on). An interesting study carried out by Yamada et al. [106] compared the osteoclastic response to different biphasic calcium phosphates depending on the β-TCP-to-HA ratio (wt %), which was changed from 100% of β-TCP to 100% of HA through 75/25 β-TCP/HA and 25/75 β-TCP/HA. Resorption lacunae were observed on pure β-TCP and 75/25 β-TCP/HA material; on the contrary, the material containing higher amounts of HA was not resorbed by osteoclasts. The authors found that the lobulated lacunae formed on the surface of biphasic ceramics are similar to those presented on the natural bone. Moreover, the high dissolution rate of pure β-TCP and the totally absent osteoclast activity on the pure HA suggest that the use of a combination of β-TCP and HA (as a biphasic material) provides a proper condition for osteoclasts to act more naturally. As new bone deposition by osteoblasts is strongly related with osteoclastic resorption during bone remodeling, biphasic calcium phosphates promote the creation of a surface similar to that of the native bone, and hence presumably the dissolution/precipitation process which occurs during the osteoclastic activity favors the formation of a chemical bond between bone apatite and similar apatite formed on the ceramic surface. By changing the proportion between HA and β-TCP, it is feasible to control the degradation rate of the bone graft and, accordingly, the bone formation. The mechanical properties of grafting materials are controlled by the amount of β-TCP and HA of the biphasic material; in particular, a material with a high percentage of β-TCP shows poor mechanical properties. Morra et al. [116] recently developed a biphasic granulate bone filler with a HA/β-TCP weight ratio of 75/25; after being implanted in rabbits, this graft elicited an excellent new bone formation without any inflammatory response. The mechanical properties were shown to depend not only on the composition but also on the geometry (e.g., the presence of macropores) and process parameters (i.e., sintering temperature and time), thus opening a broad range of possibilities for optimizing the properties of these bone grafts for use in periodontal tissue engineering. 

#### 3.3.2. Bioactive Glasses 

In bone tissue engineering, a bioactive material has been traditionally defined as a material that undergoes specific surface reactions in vitro and in vivo, promoting the formation of a HA-like layer which allows a strong bond between host bone and grafting material to occur [87,117,118]. This ability was also referred to as osteostimulation by Schepers and Ducheyne [119]. Recent studies have revealed that bioactive glasses are osteoinductive materials, too, as they can induce osteoprogenitor cells to migrate into the structure of the graft and can promote cell differentiation by affecting the gene expression of undifferentiated cells [120,121,122]. These exceptional properties have been associated with the release of therapeutic ionic species, mainly silicate and calcium ions, which can stimulate bone cells towards paths of regeneration and self-repair [123,124]. 

The most commonly-used bioactive glass with a 50-year history is the well-known 45S5 Bioglass^®^ (45SiO_2_-24.5Na_2_O-24.5-CaO-6P_2_O_5_ wt %), which exhibits a relatively low SiO_2_ content, high Na_2_O, and CaO content, and high CaO/P_2_O_5_ ratio to favor the reactivity with biological fluids [125]. This composition promotes the formation of a bone-like nano-crystalline HA layer on the surface of the glass, which can therefore firmly bond to the host bone [126,127]. 45S5 glass particulate, commercially marketed as PerioGlas^®^ (NovaBone Products LLC, Alachua, FL, USA) in dental applications, was proved able to inhibit the down-growth of epithelial cells and to promote the regeneration of alveolar bone [128,129]. Bioactive glass particles were also added to the autologous material in large infrabony defects, where the amount of grafting material needed is large, and there is the need for high mechanical properties [130]. 

More recent studies in small animal models have proposed the use of borosilicate glasses to regenerate bone as they are characterized by faster apatite-forming and bone-regenerative abilities than silicate glasses [131]; however, no studies dealing with the specific application of these glasses in periodontal tissue engineering have been reported yet in the literature. 

#### 3.3.3. Calcium Sulfate 

Calcium sulfate, which is also known as ‘plaster of Paris’, was used as a tissue augmentation material for the first time in 1892 to fill cavities caused by tuberculosis [132]. Since then, this material has been widely used in orthopedics and dentistry to fill bone defects [133]. Three different forms of calcium sulfate exist depending on the number of water molecules inside their crystalline structure, namely the anhydrate, dehydrate, and hemihydrate [134]; the last material, being quickly soluble, is typically used in medical grade products [135]. 

After being completely degraded in biological fluids, calcium sulfate leaves behind calcium phosphate deposits that stimulate bone growth [133,136]. Moreover, it has been documented that the porosity and hygroscopic properties of calcium sulfate are the key factors allowing adsorption and infiltration of platelets to stimulate the formation of bone and new blood vessels (i.e., angiogenesis). It is worth noting that no adverse reaction (e.g., immunogenicity) has been reported regarding by-products of calcium sulfate, and it could be classified as a short-term space maintainer. Calcium sulfate is preferably used in the forms of moldable paste or putty, and there are many publications dealing with its clinical effectiveness and safety in filling periodontal defect [137,138,139]. Furthermore, calcium sulfate has been used as a tentative barrier material to prevent ingrowth of soft tissues, even if its tendency to rapidly dissolve limits its efficacy in the short term [140,141]. Gitelis and Brebach also impregnated calcium sulfate pellets with antibiotics in order to develop an implantable system for local drug delivery [142]. 

If good biological properties and relative inexpensiveness have encouraged the use of calcium sulfate as a graft material for many years, the advent of other biomaterials with more controllable solubility and predictable fate in contact with blood and saliva has progressively reduced its use over the last decade [136,143]. In order to overcome the problems associated with fast resorption, calcium sulfate has also been combined with other materials, such as calcium phosphates, thereby achieving a more stable structure and a finer control on the resorption kinetics [138,139,141]. Other approaches involve the production of biphasic calcium sulfate, in which dehydrate and hemihydrate types are mixed to decrease the dissolution rate and make a rigid matrix post-implantation [144]. Today, the primary uses of calcium sulfate and its composites in dentistry and maxillofacial surgery are in the field of injectable bone fillers for sinus augmentation and alveolar bone regeneration in small periodontal defects [145].

### 3.4. Polymers 

The use of soft moldable materials in periodontal tissue engineering is a widespread practice. They can be applied as barrier membranes, hydrogels, and in combination with glass and ceramics. Natural polymers can mimic the extracellular matrix (ECM) of the bone tissue and allow cell infiltration, proliferation, and new bone formation [146]. Natural polymers possess a few properties, including elasticity, hydrophilicity, biodegradability, moldability, and biocompatibility, which are very suitable for tissue engineering applications. However, there are some disadvantages, too, including source variability, immunogenicity (if they are not pure), and poor mechanical properties (compared to bone) when used alone.

#### 3.4.1. Collagen 

**Figure 3:**
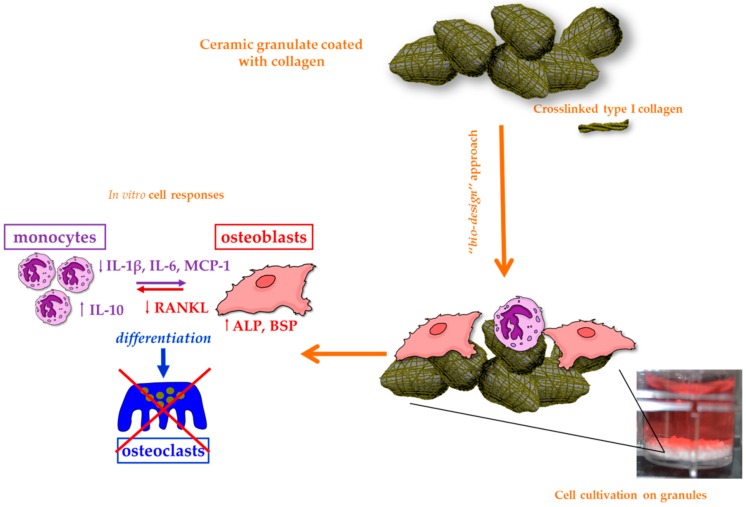
In vitro cell response of a ceramic granulate (Synergoss^®^; see also [116]) coated with a biomimetic collagen layer. This ‘bio-design’ approach improves the expression of osteogenic markers and reduces the inflammation response by macrophages.

Collagen is the most popular among the natural materials for tissue engineering since it has proven biocompatibility and ability to promote wound healing [147]. It may be extracted from several allogenic sources being the most common structural protein found in the ECM of different connective tissues such as bone, cartilage, muscle, tendon, and skin [148]. Up to now, a large number of studies have been conducted on type I collagen that is the most abundant form of collagen in the body and forms the major component of periodontal connective tissue [147,148]. Collagenous materials degrade via enzymatic lysis, and the resulting by-products are biocompatible and do not cause any inflammatory response [149]. Collagen is widely used in barrier membranes as guiding material for tissue regeneration, in hydrogel sponges and as a coating material on ceramic scaffolds or metallic dental implants to mimic the natural component of bone ECM, thus providing the underlying substrate with a biomimetic surface (Figure 3) [79,150]. The tensile strength and elastic modulus of collagen can be improved by crosslinking agents, such as (1-ethyl-3-(3-dimethylaminopropyl) carbodiimide hydrochloride) (EDC), glutaraldehyde, and tannic acid. Crosslinking of collagen fibers typically improves the mechanical properties and decreases the degradation rate, but it can have a negative impact on cell response since most of the chemical crosslinking agents are toxic [151,152]. 

#### 3.4.2. Chitosan 

Chitosan is a natural linear polysaccharide derived from chitin that is commonly extracted from the crustaceous exoskeleton and is composed of randomly distributed β–(1,4)–glucosamine and N-acetyl-D-glucosamine [153]. White mushrooms are other sources of chitosan which may be preferred to eliminate any animal-related immunogenic and ethical issues; however, the extraction process is a bit more expensive, and the yield is lower.

The widespread use of chitosan in bone tissue engineering applications and pharmaceutics is related to its attractive characteristics including biocompatibility, antibacterial activity, and the ability to promote cell adhesion, proliferation, and differentiation [154,155]. Furthermore, chitosan has a backbone similar to that of glycosaminoglycans, the major components of bone ECM [156]. After being implanted in patients with periodontitis, chitosan could reduce gingival inflammation thanks to its antimicrobial properties [157]. 

Many biomedical applications involve the combination of soft chitosan with stiff, high-strength bioceramics to produce porous foams or composite pastes [158,159]. 

Interestingly, chitosan exhibits a poly-cationic nature allowing the creation of ionic interactions with other poly-anionic materials, thus generating the so-called polyelectrolyte (PEI) hydrogel [153,160,161,162]. These properties are used to produce smart materials for controlled drug release, especially for use in the intestinal tract: in fact, this ionic bond is pH-sensitive e and, therefore, the drug release rate depends on the pH of the organ [160,163].

#### 3.4.3. Pectin 

Pectin is a natural anionic polysaccharide which is abundantly contained in citrus cell walls and apple peel by-products and consists of a poly(D-galacturonic acid) chain with partly-methoxylated carboxylic groups [164,165]. Although the use of pectin is a historical background in the food industry, it has recently found some medical applications in bone tissue engineering and controlled drug release, too [166,167]. The ionic crosslinking of pectin carboxylic groups can be achieved by calcium ions to form the so-called “egg box” structure, where a divalent cation (Ca^2+^) is bonded with different carboxylic anions [168,169]. Furthermore, a PEI structure can form as a result of the ionic interactions with poly-cationic polysaccharides, as already observed for chitosan [170]. 

The major limitation of pectin is its fast solubility in aqueous media, which causes rapid dissolution and when used as a drug carrier, a burst release of the therapeutic molecules [162]. In the attempt to overcome this problem, studies have been carried out to combine pectin with other materials, such as chitosan, in order to increase the chemical resistance in water [161,163,171,172,173]. Pectin has also been used in combination with calcium phosphate particles, since it can mimic the ECM and guide cell proliferation, or be applied as a surface coating in different biomedical applications, such as antiadhesive surgical meshes [174,175,176,177,178].

#### 3.4.4. Alginate 

Alginic acid is a natural polymer extracted from brown algae or synthesized through bacterial biosynthesis, which allows obtaining macromolecules with more controllable structure. Alginic acid is identified as a linear copolymer made of (1,4)-linked β-D-mannuronate (M) and α-L-guluronate residues (G) blocks [179]. The characteristics of the alginate material are determined by the amount of the blocks composed of consecutive G and M residues [180]. Alginate is a suitable material for biomedical applications due to its significant features including good biocompatibility, relatively low cost, and simplicity of gelation [113]. However, alginate suffers from low mechanical strength, like all other natural polymers, and need to be coupled with other materials, such as pectin, chitosan, β-TCP, or bioactive glasses [161]. Alginate hydrogels and alginate/bioactive glass composites have been experimented in periodontal tissue engineering and were found able to induce osteoblastic cell differentiation and enhance alkaline phosphatase (ALP) activity of human periodontal ligament fibroblast cells [181,182]. 

#### 3.4.5. Hyaluronic Acid 

Hyaluronic acid (or hyaluronan) is a highly-attractive material for periodontal tissue engineering since it is one of the natural glycosaminoglycans contained in the ECM of connective tissues, with excellent potential for making scaffolds for tissue regeneration [183]. Hyaluronic acid is a linear polysaccharide and, in dentistry, was shown to elicit anti-inflammatory and antibacterial effects in the treatment of periodontal diseases [184]. 

The repeating unit of hyaluronic acid is composed by d-glucuronic acid bonded to N-acetyl-d-glucosamine [185,186]. Hyaluronan exhibits hygroscopic and viscoelastic properties, which allow the material to absorb a huge amount of water while maintaining conformational stiffness and to fill the defect space, thus protecting the exposed tissue surfaces in periodontal surgery [186,187,188]. Furthermore, hyaluronic acid can act as a stable barrier against the penetration of viruses and bacteria and elicit a bacteriostatic effect, which are key features to avoid the contamination of surgical wounds by foreign pathogens and to reduce the risk of postoperative infections, thereby promoting a more predictable regeneration [189,190,191]. 

## 4. Porous Scaffolds for Bone Grafting and Periodontal Tissue Engineering 

Ideally, grafting materials for the alveolar bone should be shapable in different forms, depending on the specific application, defect size and preference of the dental surgeon, and act as a template (scaffold) for the growth of regenerated tissue in 3D [31]. Scaffolds should fill the void space, avoiding the collapse of the defect, promote cell, platelet and vessel infiltration, be easy to manage, and degrade at a rate comparable with new bone growth. The chemical composition and final morphology are considered as the main players determining scaffolds characteristics like mechanical properties, porosity, and degradability. Porosity is the crucial feature as all other parameters strongly depend on it [192]. Hence, many studies have been carried out to understand which pore size, pore distribution, and pore interconnectivity are preferable for promoting osteoblast proliferation and bone ingrowth [193]. 

The scaffolds used as bone grafts should be not only osteoconductive but also osteoinductive, thereby stimulating cells to proliferate and differentiate [194,195]. In order to achieve an optimal osteointegration, the scaffold material should mimic as close as possible the bone morphology following a biomimetic approach. Natural bone is mainly composed of HA crystals deposited in a collagen matrix (biocomposite) [196] and can exhibit either a trabecular (porosity within 50–90 vol %, pore size up to 1 mm) or a dense morphology (cortical bone, with a porosity in the range of 3 to 12 vol %) [197,198,199]. The mechanical properties of human bone broadly vary by age and depending on the harvesting site in the body [199]. 

It has been well-documented that not only total porosity but also pore size of scaffolds have a crucial role in bone formation in vitro and in vivo [193]. The morphology and porosity of the bone graft also influence the osteogenesis, the mechanical properties, and the degradation rate: typically, higher the porosity, lower the strength and elastic modulus, and faster the resorption rate (due to the higher specific surface area) [200,201].

Numerous techniques have been developed to fabricate porous scaffolds, such as gas foaming [202], salt leaching [203], phase transformation [204], freeze-drying [205], sponge replication [206] as well as the recently-developed additive manufacturing methods [207]. The processing technique depends on the specific material(s) used and should be carefully optimized to obtain an interconnected porosity with controlled pore size. 

The key role played by porosity in bone regeneration was well pointed out by Kuboki et al. using a rat ectopic model implanted with porous and dense HA particles [200]. The results showed no new bone formation on the dense particles, whereas porous particles could promote the formation of new bone tissue. Hulbert et al. [208] first defined the pore diameter of 100 μm as the lowest limit for osteogenic promotion. In this study, calcium aluminate scaffolds with different pore sizes and a constant porosity of 46 vol % were implanted in dog femurs, and no bone ingrowth was observed in the implant having pores smaller than 100 μm, whereas there was the highest new bone formation in the samples having a pore size within 150–200 μm. These results are in agreement with the diameters of the Haversian bone system, which reach values around 100–200 μm, and were then confirmed by most studies carried out in the next years. There is also a minority of works reporting no significant difference, regarding bone regeneration, between scaffolds having pores lower or higher than 100 μm [200,209,210]. As a general comment, the variability of these results can be partially justified by the different experimental conditions used, e.g., different materials, implantation site, and animals just to mention a few ones. A very comprehensive and acute analysis of these issues has been recently reported by El-Rashidy et al. [211]. Comparison between in vitro and in vivo results is even much more difficult due to the reasons mentioned above. 

The effects of porosity and pore size were evaluated in vitro using osteoblasts and mesenchymal stem cells [212,213]. Small-sized pores allow cell aggregation and inhibit cell proliferation, resulting in an increased expression of ALP and osteocalcin [214]. On the contrary, macropores allow cell proliferation since large pores enhance nutrient and oxygen transportation. Very interestingly, in vitro osteogenesis does not seem to be affected by pore size but is promoted by a low pore fraction [193]. Akay and coworkers evaluated the effect of pore size using primary rat osteoblasts and reported that the samples with smaller pores could support cell growth, proliferation, and migration better than their counterparts with large pores. Nonetheless, they observed no significant difference between the two samples regarding the mineralization [213]. 

A multitude of variables characterize the in vivo scenario: for example, osteogenesis strongly depends on the degree of vascularization, which is favored by high porosity (in contrast with in vitro observations [193]) and large pore size to allow blood vessel access and development. It was shown that small pores (below 100 μm) promote chondrogenesis before osteogenesis and low porosity, being often characterized by non-interconnected pores, does not permit nutrient transportation [215,216,217]. It has been demonstrated that high porosity and large interconnected pores promote both in vivo osteogenesis by the recruitment of osteoblastic cells, which are stimulated to migrate into the scaffold, and vascularization that is the key to favor new bone formation [193]. In this regard, porous HA scaffolds with different pore sizes were implanted subcutaneously in rats, and the results showed an increase in the ALP activity for the material with pore sizes within 300–400 μm, along with good capillary infiltration of fluids and cells [216,218].

Pore geometry also affects bone regeneration: long channels and interconnected spheroidal pores promote cell colonization and bone ingrowth, while curved pores with poor interconnections on the surface of the scaffold (like surface ‘pits’) hinder osteoblast precursor cell penetration and capillary infiltration, thus allowing bone formation only on the scaffold surface [215]. 

The degree of porosity, pore size, and pore interconnectivity are key determinants for the mechanical scaffold properties. It should be considered that, on the one hand, high porosity and large pore size result in the promotion of bone ingrowth but, on the other hand, a high void volume reduces the mechanical properties (primarily strength and stiffness) and is dangerous for the structural integrity of the scaffold [219]. 

It is essential to take measures on the degradation rate of the material(s) used to produce the scaffold; if the material has a fast degradation rate, the initial porosity should not be too high because the rapid erosion of the scaffold struts could negatively affect the mechanical and structural integrity of the implant before it is substituted by newly formed bone. On the other hand, if the degradation rate of the biomaterial is low and the mechanical strength is high, it is possible to introduce a high percentage of voids in the scaffold as the presence of channels and interconnected pores is considered as one of the main reasons for the accelerated degradation due to macrophages (via enzymatic lysis) and/or hydrolysis. 

The simultaneous presence of both micro- and macropores in the scaffold structure is recommended, as relatively small pores below 100 μm promote protein adsorption and cell aggregation, and large pores allow vascularization and new bone ingrowth [220]. Furthermore, the mechanical properties and degradation rate can be finely tailored through providing a balance between large and small pore. 

**Figure 4:**
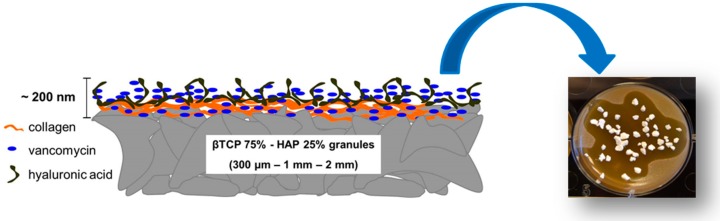
Schematic representation of a layer-by-layer surface treatment in which collagen fibrils are combined with hyaluronic acid and vancomycin. This multifunctional surface elicits a sustained antibacterial activity and a pro-osteogenic response (see also [222]).

Ceramic materials are often combined with natural polymers to control and customize the mechanical properties and the degradation kinetics [221]. Furthermore, depositing a natural protein layer (e.g., collagen and hyaluronic acid [116]) on the surface of a ceramic graft provides the scaffold with biomimetic properties, thus enhancing the osteointegration. In this regard, the deposition of the coating according to a layer-by-layer strategy could be useful to embed and then release drug molecules in situ (Figure 4).

In general, dental surgeons prefer to use moldable fillers rather than porous but rigid implants to press the material more easily into the defects [223]. Ceramic particles are either mixed with the patient’s blood or combined with natural polymers to produce an injectable composite, which can fill irregular defects or tiny voids around titanium implants that are otherwise difficult to reach. In the presence of large bone defects, however, the use of 3D porous scaffolds can be recommended to sustain the surrounding tissues and stimulate bone regeneration adequately. Iviglia et al. [206] recently developed a 3D multifunctional porous scaffold that is potentially suitable for periodontal regeneration as well as to contrast peri-prosthetic infections around dental implants. This scaffold was produced by sponge replication using HA and β-TCP powders (25: 75 wt %) and was coated with a pectin/chitosan PEI that acted as a carrier for vancomycin. Taking the advantages of this method, the authors could obtain a controlled antibiotic release, and the results of serial bacterial dilution test after one week proved that the scaffold still inhibited the growth of *Staphylococcus epidermidis*. Compressive strength was adequate for safe handling during surgery (above 1 MPa), and degradation tests showed an excellent behavior in physiological and acidic environments (mass loss <10% in the latter case, which is typical of infections). Furthermore, the PEI coating showed an anti-inflammatory response, and good proliferation of osteoblastic cells (Saos-2 cell line) was observed in vitro.

**Figure 5:**
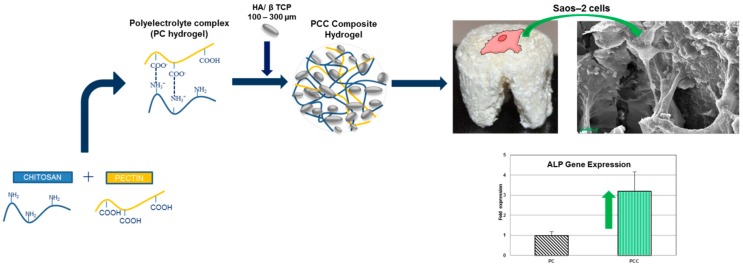
Moldable composite scaffold (pectin/chitosan hydrogel + biphasic calcium phosphate particles) for alveolar bone regeneration: the material promotes osteoblast adhesion and proliferation with a 3-fold higher ALP gene expression at one week compared to the control. Reproduced with permission from [225].

The same research group developed a moldable chitosan/pectin hydrogel scaffold reinforced with biphasic calcium phosphate particles within 100–300 µm. The polysaccharide nature of the porous hydrogel matrix mimicked the natural bone ECM, and the ceramic particles promoted high osteoblast proliferation; these characteristics, coupled with the easy adaptability to the defect dimensions, make this material highly promising for alveolar bone regeneration (Figure 5).

**Figure 6:**
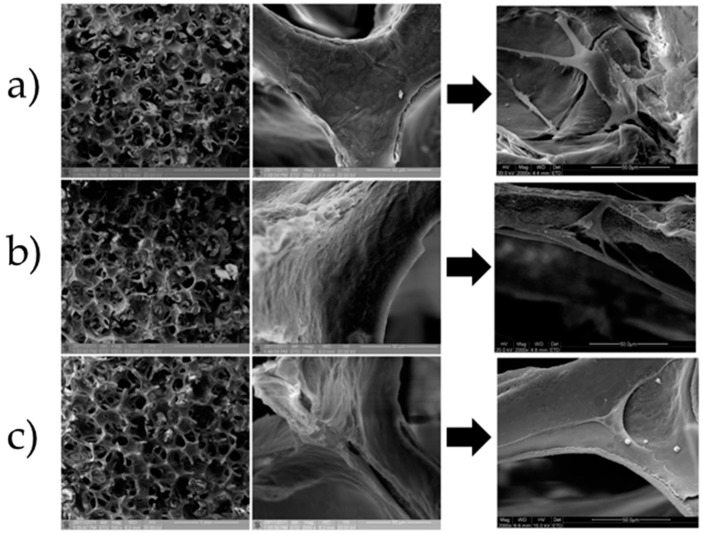
Li-doped MBGs for potential use in periodontal regeneration: SEM analysis of 0Li-MBG (**a**), 2Li-MBG (**b**), and 5Li-MBG (**c**) scaffolds before and after seeding of human periodontal ligament-derived cells (hPDLCs) on them. Reproduced with permission from [224].

**Figure 7:**
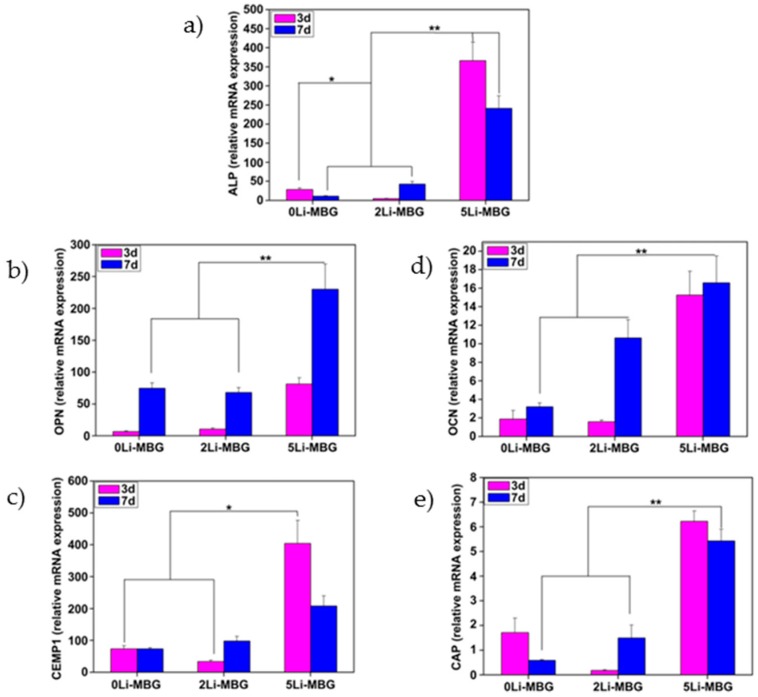
Effects of Li-doped MBG scaffolds on the expression of some of the bone-related genes such as ALP (**a**), OPN (**b**), OCN (**d**), cementum-specific markers of CEMP1 (**c**), and CAP (**e**) for hPDLCs. *: significant difference (*p* < 0.05) for the 5Li-MBG group in comparison to the other two groups at day 3. **: significant difference (*p* < 0.05) for the 5Li-MBG group in comparison to the other two groups at day 7. Reproduced with permission from [224].

Han et al. [224] also proposed to exploit the therapeutic ions released from implantable scaffolds for stimulating and directing the biological activity of the cells involved in periodontal regeneration. Specifically, lithium ions incorporated into (and then delivered from) mesoporous bioactive glass (MBG) scaffolds were shown to promote the attachment, proliferation and cementogenic differentiation of human periodontal ligament cells via activation of Wnt/b-catenin signaling pathway (Figure 6 and Figure 7). 

## 5. Guided Tissue Regeneration (GTR) Membranes 

As previously mentioned, the Melcher’s hypothesis states that certain cell types under the appropriate conditions can populate the periodontal wound and regenerate new cementum, alveolar bone, and periodontal ligament [24]. This process occurs when epithelial cells or fibroblast cells from the gingival front and connective tissue are excluded from the wound space. This goal can be achieved by developing a barrier membrane, which guides soft tissue regeneration without down-growth in the defect site [32]. Some necessary characteristics are counted for GTR membranes including compatibility with the living systems, cell exclusion, space maintenance, tissue integration, and easy use. The capabilities of separating the gingival flap from the coagulum in the wound space, of withstanding the masticatory stress and the flap tension, and of avoiding the collapse of the soft tissue while maintaining the space for the regeneration of new alveolar bone are all accounted as other important functions of the membrane, too [42]. Furthermore, bio-integration of GTR membranes ensures the stabilization of the wound and guides fibroblast cells to regenerate soft tissue without a down-growth in the periodontal defect space. As the final point, the easy use and handling of the membrane, required by dental surgeons during the operative procedures, are undoubtedly essential parameters for the GTR membrane. In the early 1980s, cellulose acetate filter was used as the first material in the role of a barrier membrane and its efficacy for the guided-tissue process was histologically proven [226]. Since then, different types of barrier materials have been developed and clinically experimented.

### 5.1. Non-Resorbable Membranes 

The high mechanical stability and the capabilities of retaining the shape and inhibiting the cell migration process (from the gingiva in the wound site) are the main advantages of non-resorbable membranes [42]. In spite of the promising results obtained from the application of this type of GTR materials, there are some critical drawbacks regarding the need for a second surgery and the possible interference with the healing process, which can be overcome only by using resorbable membranes. Polytetrafluorethylene (ePTFE) and titanium-reinforced PTFE meshes are the most commonly-implanted non-resorbable membranes.

The ePTFE (Goretex^®^) membrane was the first GTR membrane approved for clinical use in the 1990s [227]. ePTFE is chemically identical to PTFE and exhibits a porous microstructure that allows connective tissue ingrowth. ePTFE membranes for GTR have a typical bi-layered structure: the first layer, which is porous (90 vol %) and around 1-mm thick, can promote cell ingrowth, whereas the second layer (thickness 0.15 mm, porosity 30 vol %) acts as a space provider for regeneration, inhibiting the epithelial cell down-growth and giving structural stability [228,229]. Goretex^®^ membrane possesses an exceptional inertness as it does not elicit any foreign body reaction. Several clinical trials have been carried out on this material, and histological observations revealed that ePTFE membrane could lead to significant periodontal regeneration after a three-month healing period [228,230]. Some other works, however, report no significant difference between the results obtained from treatment with ePTFE membrane and the conventional debridement therapy with open flap. Moreover, some reports stated that there are some additional complications associated with ePTFE GTR membranes, like pain and purulence, along with additional costs for a second surgery if needed [231]. 

The ePTFE membrane can also be reinforced with a titanium mesh between the two layers to increase the mechanical strength and the supporting function to tissues [42,232]. It has been shown that the addition of a titanium mesh to the membrane can be useful for achieving the better positioning under the flap by the dental surgeon and for increasing the stability of the membrane, thus preventing defect collapse. Although the microporosity of the membrane avoids cell ingrowth, it can permit fluid infiltration. The results of animal experiments indicated relevant cementum and bone regeneration [233,234]; however, no difference was observed between titanium-reinforced and titanium-free ePTFE GTR membranes in clinical trials [232,235]. Furthermore, the main disadvantage is the increased risk of exposure due to titanium stiffness, which is associated with a more complex secondary surgery to remove the implant [236]. 

The future of periodontal tissue regeneration is the development and use of biomaterials which can degrade during tissue formation without the need for being removed by any further surgical procedure, thereby reducing patient’s pain, stress, and hospitalization costs. 

### 5.2. Resorbable Membranes

Owing to the need for a second surgery to remove non-resorbable membranes, in the last decade, the demand for bioabsorbable membranes with comparable and in some aspects better clinical outcome became urgent. Resorbable membranes were thought suitable to reduce the patient’s overall discomfort and, if provided with bioactive properties, to accelerate tissue healing compared to non-resorbable ones [42]. General disadvantages of these materials include the only partly predictable resorption rate, due to the different degradation processes that could take place (enzymatic or by hydrolysis), and the risk of eliciting local inflammation due to degradation [32]. Resorbable membranes can be obtained from natural sources or by synthetic procedures. Natural membranes have the advantages of being highly biocompatible and potentially bioactive, but typically lack mechanical properties and tend to degrade very rapidly in vivo [42,237]. On the other hand, synthetic materials show a more predictable behavior as their degradability and mechanical properties can be tuned by acting on the composition. However, this may result in a foreign body reaction. 

As aforementioned, the periodontal connective tissue is mostly made of type I collagen that has been widely used in periodontology due to low immunogenicity, ability to attract and activate periodontal ligament/gingival fibroblast cells, and hemostatic properties [150]. The interaction with various cell types demonstrates the bioactivity of collagen and its potential to augment tissue thickness during the healing process (Table 1). Up to now, some collagen-based membranes have been commercially produced, such as BioOss^®^ membrane (Geistlich Pharma, Wolhusen, Switzerland)), OsteoBiol^®^ (Tecnoss, Giaveno, Italy), Zimmer BioMend^®^ and BioMend Extended^®^ (Zimmer, Frankfurt, Germany) and Lyoplant^®^ (Braun AG, Melsugen, Germany). The behavior of the collagen GTR membranes is determined by the source of the collagen and on the process used to obtain it [238]. 

Sources such as skin, tendons, or bovine/porcine pericardium are usually used for collagen extraction [150]. According to the European Union’s guidelines, collagen extracted from animal sources needs to be chemically purified to eliminate all viral and bacterial contaminants. In this regard, the antigenicity of collagen should be eliminated by removing lipid and non-collagenous protein remnants. Sequential segment analysis, biocompatibility, and sterility are checked step by step [32]. The most common post-purification modification process of collagen is the chemical crosslinking, which improves its mechanical strength and reduces its degradation rate and water uptake capacity. However, this process is usually associated with increased cytotoxicity due to the possible presence of crosslinking traces inside the collagen network [152]. Degradation of collagen is due to the action of the enzyme collagenase that cuts the collagen chain and transforms it into gelatin, which is then degraded via amino acid gelatinases [32,150]. Based on the source and the crosslinking processes, the resorption time of collagen GTR membrane varies from 1 to 6 months [239,240]. Non-resorbable ePTFE GTR membrane was compared to collagen membrane, and the results showed that the latter stimulates the proliferation of gingival fibroblasts and the ECM synthesis in a significantly higher amount [235]. Wang et al. observed a better osteoblast attachment on collagen surfaces while the collagen membrane remained intact and prevented the apical proliferation of epithelial cells [241]. Animal studies using BioOss^®^ revealed that the collagen-based membrane could regenerate the periodontal tissue, and the material resorbed after eight weeks. Also, during the resorption, a small inflammatory zone was visible around the implant, which finally disappeared after total resorption [79,239].

The main drawbacks of collagen membranes include limited toughness and low space maintenance [32,242]. Therefore, there is still the need for developing a man-made material that can swell and fill the irregular shape of the gingival flap in order to stabilize the membrane; moreover, this capability should be coupled with antiadhesive properties which guide epithelial regeneration without eliciting inflammatory reactions and preventing cell in-growth in the wound defect. Organic aliphatic thermoplastic polymers—such as poly(lactic acid), poly(glycolic acid), and their copolymers—are the most commonly-used synthetic materials in GTR. The great advantage of using synthetic polymers concerns the ease of customizing some properties, such as degradation kinetics and mechanical properties, by varying the length of the chain and the amount of lactide or glycol units [243]. The most important drawback associated with man-made materials is the significant increase of pH values arising from the by-products released during the hydrolysis, which can cause cytotoxic effects [244]. Furthermore, bulk degradation of these materials is another limitation as it can lead to decreased mechanical stability and interfere with periodontal regeneration. There are some commercial synthetic products for GTR available on the market; historically, the first of them to appear on the market in the 1990s was Guidor^®^ (Sunstar Americas Inc., Schaumburg, IL, USA), which is a bilayer membrane made of poly(lactic acid) and citric acid ester acetyl tributylcitrate. The outer layer of Guidor^®^ has rectangular perforations in which new tissue can grow, while the internal layer has smaller circular perforations and outer space to maintain the distance between the membrane and the root surface [42,245]. An internal spacer between the two layers promotes tissue ingrowth. The results of animal experiments revealed that complete resorption of the membrane occurs within 12 weeks, even if the immune system initially detected the membrane as a foreign body. Over the years, a large number of other products have been developed, such as Resolute^®^ (W.L. Gore & Associates, Flagstaff, AZ, USA), made of poly(lactic-*co*-glycolic acid) co-polymer reinforced with polyglycolide fibers [246], Vycril Periodontal Mesh^®^ (Ethicon, Somerville, NJ, USA), made of a co-polymer of lactide and glycolide [247], and Atrisorb^®^ (Tolmar Inc., Fort Collins, CO, USA), which is produced in flowable poly(lactic acid) and is ready to use after exposure in saline solution for 6 minutes [248]. All the mentioned membranes have been evaluated both in vitro and in vivo, and the results have revealed their great potential regarding the stimulation of periodontal regeneration, although many of them elicit foreign-body reaction associated with the release of acidic by-products [42]. In addition to the above-mentioned polyester membranes, polyurethane membranes which degrade via enzymatic lysis have also been proposed [249]. Animal studies showed that these membranes could elicit inflammatory responses and their degradation was slower compared to poly(lactic acid) [250]. Cell response to polyurethane membrane obtained from plant-derived oil was found similar to that of PTFE non-resorbable membrane [230].

## 6. Growth Factors in Periodontal Tissue Engineering

The incorporation of bioactive molecules into periodontal biomaterials and scaffolds and their local and controlled release over time has been proposed as a valuable approach to obtain osteoinductive implants [251]. Two different procedures to embed growth factors are available, i.e., either during the preparation of the material [252] or after the fabrication [253,254]. Platelet-rich growth factor (PDGF), bone morphogenetic proteins (BMPs), and enamel matrix derivatives (EMDs) are among the most potent biomolecules for accelerating the periodontal wound repair in preclinical and clinical studies [251,255,256,257]. Growth factors have the potential of stimulating the interaction between mesenchymal stem cells (MSCs) and epithelial stem cells during tooth formation, along with all the following processes such as collagen formation, mineralized matrix deposition, and fibroblast proliferation [251,258]. The morphology of the scaffold where signaling molecules are incorporated is fundamental to achieve an extended and effective release. The incorporated molecules are released according to a diffusion mechanism which strongly depends on the porosity—especially the pore interconnectivity—of the material. The degradation properties of the graft or GTR membrane also affect the release rate of growth factors, which might be faster because of the high solubility of the matrix. Furthermore, the way in which the carrier degrades—either by surface or by bulk degradation, resulting in a controlled or burst release, respectively—plays a key role, too [259,260,261]. The research and development process addressed to produce a commercial product containing growth factors is expensive and difficult to regulate; however, it is clear that growth factors have a significant impact on tissue engineering outputs which deserves to be explored. 

### 6.1. Platelet-Rich Growth Factor (PDGF) 

PDGF is the main growth factor involved in wound healing, and there is a lot of in vitro and in vivo studies showing its ability to enhance the proliferation and migration of periodontal ligament cells. PDGF is naturally made of the conjugation of polypeptides of growth factor-BB and growth factor-AA, which encoded by two different genes. It has been demonstrated that all isoforms have an effect on cell proliferation in vitro [262,263]. It has been shown that PDGF has a chemotactic effect, by which it can promote collagen synthesis, and stimulates hyaluronate synthesis by gingival fibroblasts and fibroblast proliferation. Furthermore, if added to a culture with osteoblast-like cells, PDGF can regulate the ALP and osteocalcin expression [264]. Lynch et al. [265] applied PDGF in combination with the insulin-like growth factor-1 (IGF-1) in dogs, and the results demonstrated great effectiveness in periodontal regeneration. Furthermore, the results of clinical trials revealed that the synergistic effect of these two growth factors could lead to the stimulation of bone regeneration in periodontal defects in humans, too [266]. Even if used alone, PDGF can significantly stimulate the formation of new cementum and the production of collagen [267]. Molecular cloning and large-scale purification have permitted the production of recombinant human PDGF, which has been mixed with β-TCP and made commercially available to clinicians (GEM 21s^®^, Osteohealth, Shirley, NY, USA) [268]. 

### 6.2. Bone Morphogenetic Proteins (BMPs)

BMPs belong the superfamily of transforming growth factor-beta (TGF-β) [251,268]. They are abundant proteins in the bone tissue and are produced by many cell types such as osteoblasts. BMP-2, -4, and -7 are typically retained in bone allografts that are therefore osteoinductive and can actively influence cell behavior in vivo. Some animal and human studies have given convincing evidence of the great potential of BMPs in periodontal regeneration with a significant acceleration of alveolar bone healing [269,270,271]. BMPs encourage the division and chemotaxis of undifferentiated mesenchymal stem cells (MSCs) and osteoblast precursors through activating the expression of genes involved in bone formation (e.g., osteocalcin and ALP) [272]. The production of synthetic BMPs is very expensive, which limits their incorporation and use in synthetic biomaterials. 

### 6.3. Enamel Matrix Derivatives (EMDs) 

The ideal purpose of dental surgeons is achieving periodontal regeneration by mimicking the natural process that takes place during tooth formation. It has been demonstrated that the Hertwig’s cells are responsible for the secretion of enamel matrix proteins during the development of a nascent root and periodontal tissue. These proteins are then deposited onto the root surface and provide an initial and essential step that influences the migration of the surrounding cells and the formation of cementum, periodontal ligament, and alveolar bone [273,274]. The observation of this protein layer between the peripheral dentin and the cementum resulted in the development of EMDs in the form of purified acid extracts of proteins from pig enamel matrix (Emdogain^®^, Strauman AG, Basel, Switzerland) [275,276]. The main component of EMD is amelogenin, which is a specific ECM protein directing the mineralization of enamel [257]. Amelogenin, under physiological conditions, is assembled into nanospheres to form ECM, which during enzymatic degradation by metalloproteinases releases bioactive peptides for weeks [43,258]. This process stimulates new bone formation, root resorption, and wound healing. EMD can mimic odontogenesis by the recruitment and stimulation of cementoblasts to form the root cementum on the root surface [277]. Then, the newly-formed root cementum will lead to the regeneration of periodontal fibers and alveolar bone. Emdogain^®^ was clinically applied for the first time in 1997 [276], and it is currently the unique product on the market that, used alone, has the potential for triggering significant regenerative responses in periodontal ligament cells. Cambini et al. [278] recently highlighted the easiness of application of amelogenin, its persistence in situ for two weeks, and their exceptional osteoinductive capability, which made unnecessary the use of barrier materials. A systematic literature review conducted by Batistin Zanatta et al. [279] also suggests that EMDs are associated to better clinical outcomes compared to conventional treatments, but the heterogeneity and experimental differences of the studies published in the literature make definite conclusions difficult to draw.

## 7. Gene Therapy Approach in Periodontology

The critical drawbacks associated with the local delivery of growth factors include their short biological half-life in vivo as well as high cost [256,280]. Even most importantly, using a high dosage of bioactive molecules is needed to promote tissue regeneration, which could lead to unpredictable reactions and side effects; hence, an alternative approach to the local release of growth factors is the use of gene therapy for periodontal regeneration [280]. Gene therapy involves the insertion of the genes of interest into an individual’s cells to obtain the desired functions, i.e., in most cases, upregulation of the expression of a specific growth factor [38,255,281]. For this purpose, two main strategies have been proposed and developed including (i) in vivo technique, in which the gene vector is directly inserted into the target site [280,282], and (ii) ex vivo technique, in which selected cells are harvested, expanded, genetically transduced, and eventually re-implanted [283].

Gene therapy has been applied for the upregulation of the expression of PDGF [284,285,286] and BMPs [287]. In the in vivo technique the gene of interest is directly delivered in the body, thus altering the normal expression of the target cells. On the contrary, the ex vivo technique involves the use of an adenovirus vector to introduce the genetic material into the target cells that have been harvested by a biopsy; eventually, transfected cells are re-implanted in the periodontal defect [256,288]. In spite of the great potential of these techniques, there are several concerns about the safety of the adenovirus vector that still currently limit the clinical applicability of the gene therapy approach [281,289].

## 8. Nanobiomaterials and Functionally-Graded Implants: The Last Frontiers in Periodontal Tissue Engineering

Periodontal regeneration involves a set of complex tissues and structures in and around the tooth; hence, an ideal biomaterial-driven approach should include a functionally-graded scaffold where the chemical composition and 3D architecture of each layer (or ‘compartment’) match the fine organization, biochemical composition, and mechanical properties of the tissues to regenerate. This valuable strategy is in its beginning in the whole field of tissue engineering—not restricted to dentistry—and shows a great promise for the 21st-century regenerative medicine. The existing examples of multi-layer scaffolds for multi-tissue regeneration often involve the use of nanomaterials, which can more closely reproduce the fine characteristics of the structure to regenerate [290]. 

Sowmya et al. [291] recently reported the development of a 3-layer scaffold for the simultaneous regeneration of cementum, alveolar bone, and periodontal ligament. Specifically, this scaffold was structured as follows: chitosan/poly(lactic-*co*-glycolic acid) (PLGA)/nano-sized bioactive glass layer loaded with cementum protein 1 (CEMP1) for the cementum regeneration, chitosan/ PLGA layer loaded with fibroblast growth factor 2 for periodontal ligament regeneration, and chitosan/PLGA /nano-sized bioactive glass layer loaded with platelet-rich plasma (PRP) growth factors for bone regeneration. Histological and tomographic evaluations showed that the implantation of this scaffold in rabbits led to complete periodontal healing and new alveolar bone deposition after three months.

Incorporation of growth factors further stimulating tissue regeneration in nanomaterial-based constructs was also investigated. Zhang et al. [292] fabricated MBG/silk fibroin scaffolds incorporating BMP-7 and/or PDGF-B adenovirus and implanted them in dogs. The scaffolds loaded with PDGF-B adenovirus were able to partially regenerate the periodontal ligament while those loaded with BMP-7 primarily improved new alveolar bone formation. The synergistic combination of these two growth factors promoted periodontal healing by allowing up to two times greater regeneration of the periodontal ligament, alveolar bone, and cementum as compared to each adenovirus used alone.

Chen et al. [293] used a poly(ε-caprolactone) (PCL)-based multiphasic scaffold, enriched with type I collagen, as a carrier to release CEMP1-loaded poly(ethylene glycol) (PEG)-stabilized amorphous calcium phosphate nanoparticles. After implantation in rats for eight weeks, these scaffolds were shown able to stimulate cementum-like tissue formation but little new bone regeneration. 

None of these scaffolds was experimented in humans yet, probably due to the lack of clear evidence about the effects of growth factors in the long term and the fate of nanomaterials used in vivo, as well as the high cost of the devices.

## 9. Discussion and Conclusions

Periodontium refers to the specialized tissues that both surround and support the teeth, maintaining them in the maxillary and mandibular bones [25,26]. Tooth loss is a possible consequence of trauma or periodontal disease, such as gingivitis, periodontitis, or tissue decay [45]. The scope of periodontal tissue engineering is to regenerate the tooth’s supporting tissue through a combination of proper biomaterials, which stimulate cells and signaling molecules to produce new healthy tissue [8]. Many advances have been made in the last decade in the regeneration of complex periodontal and alveolar bone defects [268]. Research efforts in polymeric and ceramic scaffolding systems for cell, protein, drug, and gene delivery have led to the development of complex and often multifunctional implants with a predictable response. In the research world, there is still some debate as to the best treatment modality for obtaining periodontal regeneration [6]. Some groups advocate the use of bone replacement grafts alone, whereas others suggest that a guided tissue membrane (GTR) alone might be sufficient, and still others recommend a combination of both. Tobon et al. [294] conducted a study comparing three different treatment modalities for achieving periodontal regeneration and GTR after endodontic surgery: one control group without bone graft and membrane, one group treated with bone graft alone, and another group treated with both bone graft and membrane. They used a non-resorbable material as a membrane and HA as a bone graft. The results showed that the best periodontal regeneration was assessed through the combination of both membrane and bone graft. The worst results were obtained in the control group, where no membrane nor graft were used. Yoshikawa and co-workers [295] compared the histological outcome of different types of membranes—i.e., non-resorbable ePTFE, resorbable PLGA, and resorbable collagen membrane—and found that the greatest amount of bone regenerated was achieved using non-resorbable membranes. Another group, comparing an open flap debridement with a bone allograft and a bone allograft with a collagen membrane in an animal study, showed similar results in terms of bone formation in all cases [296]. The studies mentioned above showed different and even contrasting results, and each of them suggests different approaches in order to achieve an excellent periodontal regeneration. In general, case selection is very important to the success of regenerative strategies, which might explain some of the inconsistencies in the literature [6]. Factors that affect clinical success can be related to the specific patient, specific disease and healing categories. The success of a surgical procedure involving the use of a bone grafting material, a GTR membrane or else a combination of both, also depends on good plaque control, compliance, non-smoking, anti-infective therapy, and systemic health [297,298,299]. Furthermore, also during the surgical procedures, there may be additional variables which could affect the results of the regeneration process, such as the possible infection of the implanted material, which could cause peri-prosthetic infection.

In summary, periodontal regeneration still remains a partially unmet challenge. The incorporation of growth factors in periodonatal bioamterials and scaffolds is indeed a valuable strategy to improve regeneration, but biomolecules are typically expensive, which makes them accessible just to a minority of patients, and can elicit unpredictable side effects even at low dosage. Gene therapy has opened up new horizons to treat congenital dental diseases in individuals and their offspring; however, viral vectors used in these methods could elicit immune responses with irreversible damages. Loading and controlled release of therapeutic ions, which can stimulate the genes of cells towards paths of targeted tissue regeneration and self-repair, could be a highly-attractive alternative deserving investigation in the near future. Furthermore, the development of functionally-graded scaffolds mimicking the composition and microstructural organization of the tissues to regenerate represent a key step towards the simultaneous healing of multiple periodontal tissues.

## Figures and Tables

**Table 1:** Selected examples of commercially available collagen-based membrane for GTR

Commercial Name	Company	Sources	Cross-Linking Agent	Main Components	Resorption Rate
BioMend	Zimmer, Frankfurt, Germany	Bovine tendon	Formaldehyde	100% type I collagen	6–8 weeks
BioMend-Extend	Zimmer, Frankfurt, Germany	Bovine tendon	Formaldehyde	100% type I collagen	18 weeks
Periogen	Collagen Inc., Palo Alto, CA	Bovine dermis	Glutaraldehyde	Type I and III collagen	4–8 weeks
Paroguide	Coletica, Lyon, France	Clafskin	DPPA	96% type I collagen and 4% chondroitin-4-sulfate	4–8 weeks
Biostite	Coletica, Lyon, France	Calfskin	DPPA	88% HA 9.5% type I collagen, and 2.5% chondroitin-4-4sulfate	4–8 weeks
BioGide	Geistlich, Wolhusen, Switzerland	Porcine dermis	None	Type I and III collagen	24 weeks
Tissue Guide	Koken Co., Tokyo, Japan	Bovine dermis + tendon	HMDIC	Atelocollagen (I°) + tendon collagen	4–8 weeks
BioBar	Colbar Research & Dev. Ltd., Ramat-Hasharon, Israel	Bovine dermis	N/A	100% type I collagen	6–8 moths
Osteobiol	Tecnoss srl, Giaveno, Italy	Heterologous mesenchymal tissue	None	100% equine collagen	8 weeks
